# A chip-integrated comb-based microwave oscillator

**DOI:** 10.1038/s41377-025-01795-0

**Published:** 2025-04-30

**Authors:** Wei Sun, Zhiyang Chen, Linze Li, Chen Shen, Kunpeng Yu, Shichang Li, Jinbao Long, Huamin Zheng, Luyu Wang, Tianyu Long, Qiushi Chen, Zhouze Zhang, Baoqi Shi, Lan Gao, Yi-Han Luo, Baile Chen, Junqiu Liu

**Affiliations:** 1 International Quantum Academy, Shenzhen, 518048 China; 2 School of Information Science and Technology, ShanghaiTech University, Shanghai, 201210 China; 3 Qaleido Photonics, Shenzhen, 518048 China; 4 Hefei National Laboratory, University of Science and Technology of China, Hefei, 230088 China

**Keywords:** Integrated optics, Microwave photonics, Solitons, Nonlinear optics

## Abstract

Low-noise microwave oscillators are cornerstones for wireless communication, radar and clocks. The employment and optimization of optical frequency combs have enabled photonic microwave synthesizers with unrivalled noise performance and bandwidth breaking the bottleneck of those electronic counterparts. Emerging interest is to use chip-based Kerr frequency combs, namely microcombs. Today microcombs built on photonic integrated circuits feature small size, weight and power consumption, and can be manufactured to oscillate at any frequency ranging from microwave to millimeter-wave band. A monolithic microcomb-based microwave oscillator requires integration of lasers, photodetectors and nonlinear microresonators on a common substrate, which however has still remained elusive. Here, we demonstrate the first, fully hybrid-integrated, microcomb-based microwave oscillator at 10.7 GHz. The chip device, powered by a customized microelectronic circuit, leverages hybrid integration of a high-power DFB laser, a silicon nitride microresonator of a quality factor exceeding 25 × 10^6^, and a high-speed photodetector chip of 110 GHz bandwidth (3 dB) and 0.3 A/W responsivity. Each component represents the state of the art of its own class, yet also allows large-volume manufacturing with low cost using established CMOS and III-V foundries. The hybrid chip outputs an ultralow-noise laser of 6.9 Hz intrinsic linewidth, a coherent microcomb of 10.7 GHz repetition rate, and a 10.7 GHz microwave carrier of 6.3 mHz linewidth – all the three functions in one entity occupying a footprint of only 76 mm^2^. Furthermore, harnessing the nonlinear laser-microresonator interaction, we observe and maneuver a unique noise-quenching dynamics within discrete microcomb states, which offers immunity to laser current noise, suppression of microwave phase noise by more than 20 dB, and improvement of microwave power by up to 10 dB. The ultimate microwave phase noise reaches −75/−105/−130 dBc/Hz at 1/10/100 kHz Fourier offset frequency. Our results can reinvigorate our information society for communication, sensing, imaging, timing and precision measurement.

## Introduction

Low-noise microwave oscillators are ubiquitously deployed in our information society for communication, sensing, spectroscopy and timing. A particularly important frequency band is the microwave X-band (8 to 12 GHz), dedicated for radar, wireless networks, and satellite communication. Currently, a paradigm shift is ongoing which utilizes photonics to synthesize X-band microwaves^1–9^. Compared to the electronic counterparts, the photonic microwave generation enables unrivalled performance, particularly in noise and bandwidth.

Among all photonics-based approaches, optical frequency combs^10–12^, which coherently link radio- or microwave frequency to optical frequency, have enabled microwaves with unrivalled spectral purity (noise) by leveraging optical frequency division (OFD)^1–3^. Optical-clock-referenced OFD^13^ has created 10 GHz microwaves with 10^-16^ fractional frequency instability at 1 s, two orders of magnitude more stable than the caesium fountain clocks defining the SI second. Currently, by virtue of high-*Q* optical microresonators and harnessing the Kerr nonlinearity, an emerging paradigm is to develop and apply microresonator-based Kerr frequency combs, i.e. “microcombs”^14–22^ that have small size, weight and power consumption. In particular, exploiting recent breakthroughs in fabrication of ultralow-loss photonic integrated circuits (PIC)^23–25^, optical microresonators can now be constructed on a plethora of integrated material platforms^26,27^. These PIC-based microresonators have permitted chip-level microcombs with diverse merits and functions. Notably, latest endeavors have demonstrated microcomb-based OFD^28–32^. Despite, these OFD setups are still bulky, and require sophisticated locking techniques, power amplifiers and external references. Therefore, a free-running microcomb-based microwave oscillator that is fully integrated at chip scale is still beneficial, though the phase noise can be compromised.

**Fig. 1:**
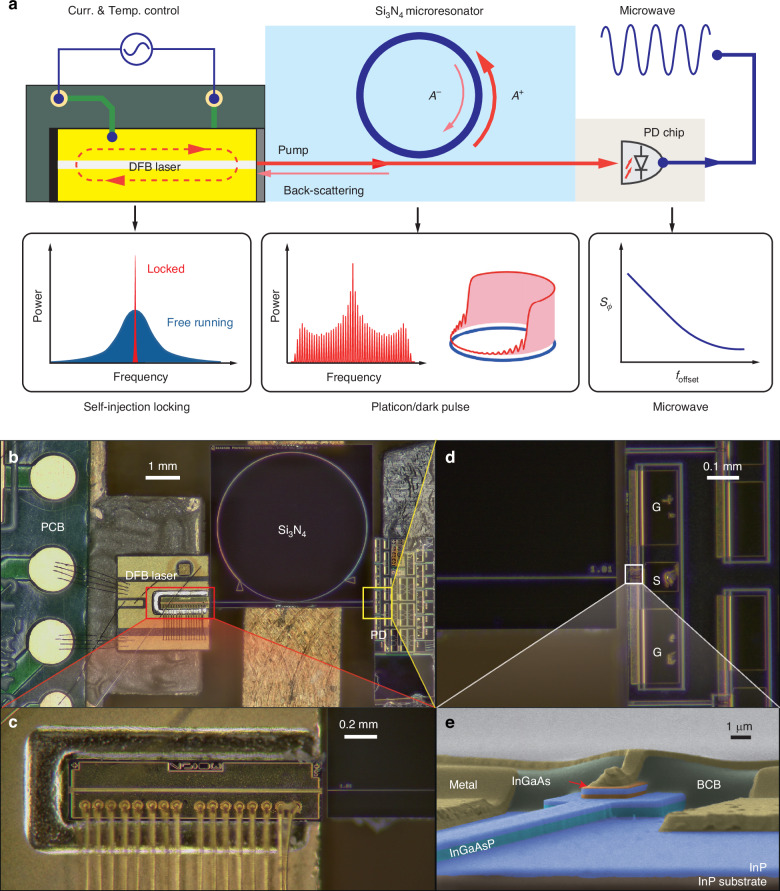
Schematic and images of the hybrid, microcomb-based, photonic microwave oscillator chip. **a** Schematic of the chip device. The microelectronic circuit supplies current to the DFB laser and stabilizes its temperature. Via edge-coupling, the CW light from the laser enters the ${{\rm{Si}}}_{3}{{\rm{N}}}_{4}$ microresonator (${A}^{+}$). Exploiting the optical back-scattering (${A}^{-}$) from the ${{\rm{Si}}}_{3}{{\rm{N}}}_{4}$ microresonator to the laser, laser self-injection locking occurs that significantly narrows the laser linewidth. Simultaneously a circulating platicon/dark pulse stream is formed in the microresonator. The output pulse stream is received by the PD chip that outputs a microwave carrier at the pulse repetition rate. ${S}_{\phi }$, microwave phase noise. ${f}_{{\rm{offset}}}$, Fourier offset frequency. **b** Photo of the hybrid chip device and individual components. **c** Zoom-in image showing the DFB laser wire-bonded to the PCB and edge-coupled to the ${{\rm{Si}}}_{3}{{\rm{N}}}_{4}$ microresonator chip. **d** Zoom-in image showing the PD chip edge-coupled to the ${{\rm{Si}}}_{3}{{\rm{N}}}_{4}$ chip and connected by a ground-signal-ground (GSG) probe for microwave signal output. **e** False-colored SEM image showing PD’s multilayer structure. InGaAs, indium gallium arsenide. InGaAsP, Indium gallium arsenide phosphide. InP, Indium phosphide. BCB, benzocyclobutene, a kind of resin

Here, we demonstrate the first, fully hybrid-integrated, microcomb-based, photonic microwave oscillator. The chip device leverages hybrid integration^33–35^ of a distributed-feedback (DFB) laser chip, a high-*Q* silicon nitride (Si_3_N_4_) microresonator chip, and a photodetector (PD) chip. The schematic and components are depicted in Fig. 1. The DFB laser chip is driven and stabilized by a microelectronic circuit, and emits CW light at 1550 nm. Via edge-coupling, the light enters the ${{\rm{Si}}}_{3}{{\rm{N}}}_{4}$ microresonator of 10.7 GHz free spectral range (FSR), where a coherent platicon microcomb of 10.7 GHz repetition rate is formed. The output light from the ${{\rm{Si}}}_{3}{{\rm{N}}}_{4}$ microresonator is delivered to the PD chip. Upon photodetection of the microcomb’s repetition rate^4–6^, the PD outputs a microwave carrier of 10.7 GHz and its harmonics. The entire device occupies a footprint of only 76 mm^2^.

## Results

### Characterization of individual components

#### Laser

We use a commercial DFB laser as shown in Fig. 1(b, c). The laser outputs 160 mW CW light at 1549.9 nm with 500 mA laser current. The free-running DFB laser exhibits 55 mA current at laser threshold, transverse-electric (TE) polarization, and 1 nm wavelength tunability over a laser current up to 500 mA. The laser’s temperature is stabilized at 30 °C using a thermo-electric cooler (TEC). A printed circuit board (PCB) provides driving current and temperature stabilization to the laser chip. The laser chip is edge-coupled to the ${{\rm{Si}}}_{3}{{\rm{N}}}_{4}$ chip^34–36^. The laser output waveguide is aligned to the inverse taper of the ${{\rm{Si}}}_{3}{{\rm{N}}}_{4}$ bus waveguide and side-coupled to the microresonator. The coupling efficiency between the laser chip and the ${{\rm{Si}}}_{3}{{\rm{N}}}_{4}$ chip is measured as 23%, i.e. with 6.4 dB insertion loss. Extra characterization data are found in Supplementary Materials Note 1. This loss value can be reduced in the future using optimized inverse taper that matches the laser output mode.

#### Microresonator

The Si_3_N_4_ chip contains a high-*Q* microresonator of 10.7 GHz FSR, as shown in Fig. 1b. A high *Q* is critical for microcomb generation, since the power threshold ${P}_{0}$ for Kerr parametric oscillation is ${P}_{0}\propto 1/({D}_{1}{Q}^{2})$, where ${D}_{1}/2\pi=10.7\,{\rm{GHz}}$ is the FSR^14,15^. We fabricate Si_3_N_4_ waveguides of 300 nm thickness using a deep-ultraviolet (DUV) subtractive process on 150-mm-diameter (6-inch) wafers^37^. The optimized fabrication process is described in Supplementary Materials Note 2. We choose $300\,{\rm{nm}}$ ${{\rm{Si}}}_{3}{{\rm{N}}}_{4}$ thickness for the following reasons. First, since high-quality ${{\rm{Si}}}_{3}{{\rm{N}}}_{4}$ films deposited via low-pressure chemical vapor deposition (LPCVD, as used in our case) are prone to crack due to intrinsic tensile stress^38^, ${{\rm{Si}}}_{3}{{\rm{N}}}_{4}$ films with thickness below $300\,{\rm{nm}}$ are free from cracks. Second, $300$-${\rm{nm}}$-thick ${{\rm{Si}}}_{3}{{\rm{N}}}_{4}$ fabrication process is currently established as a standard process in nearly all CMOS foundries worldwide^39,40^.

**Fig. 2:**
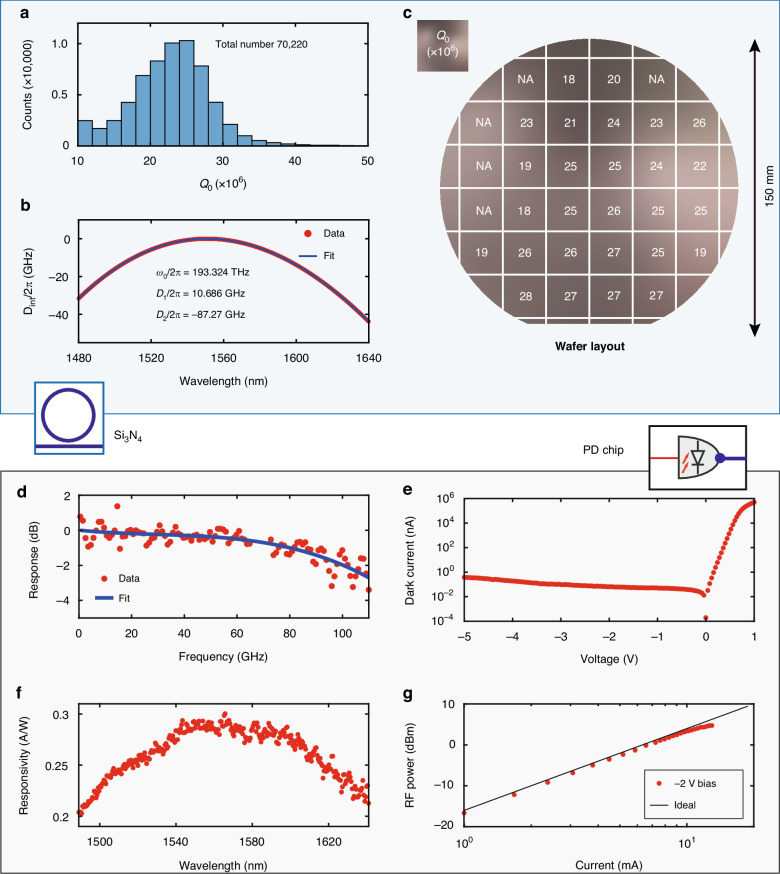
Characterization of ${{\bf{Si}}}_{{\bf{3}}}{{\bf{N}}}_{{\bf{4}}}$ microresonators and the photodetector chip. **a** Histogram of $70,220$ measured intrinsic quality factors ${Q}_{0}$ from twenty-seven ${{\rm{Si}}}_{3}{{\rm{N}}}_{4}$ chips on a $6$-inch wafer. The most probable value is ${Q}_{0}=25\times {10}^{6}$. **b** Integrated dispersion profile ${D}_{\mathrm{int}}/2\pi$ of the $10.7$-${\rm{GHz}}$-FSR ${{\rm{Si}}}_{3}{{\rm{N}}}_{4}$ microresonator. Red dots are measured data, and the blue line is the polynomial fit. The reference frequency ${\omega }_{0}/2\pi =193.324\,{\rm{THz}}$ corresponds to the pump DFB’s frequency. ${D}_{1}/2\pi =10.686\,{\rm{GHz}}$ is the microresonator FSR. ${D}_{2}/2\pi =-87.27\,{\rm{kHz}}$ is the normal GVD. **c** The most probable values ${Q}_{0}$ of the ${{\rm{Si}}}_{3}{{\rm{N}}}_{4}$ chips at different places uniformly across the $6$-inch wafer. In most places ${Q}_{0}\ge 20\times {10}^{6}$ is found, showing high yield of the ${{\rm{Si}}}_{3}{{\rm{N}}}_{4}$ fabrication process. NA, not available. **d** Frequency response of the $3\times 15\,{{\rm{\mu }}{\rm{m}}}^{2}$ PD chip. Red dots are measured data, and the blue line is the polynomial fit. **e** Measured dark current versus bias voltage of the PD chip. Negative bias voltage leads to dark current below $1\,{\rm{nA}}$. **f** Measured responsivity versus wavelength of the PD chip. **g** RF power versus the AC current of the PD chip. Measured data (red dots) are aligned with the ideal case (black line)

We characterize $70,220$ resonances in the fundamental TE mode from twenty-seven chips uniformly distributed over the $6$-inch wafer, using a vector spectrum analyzer^41^ covering $1480$ to $1640\,{\rm{nm}}$ (see Materials and methods). The waveguide width of the ${{\rm{Si}}}_{3}{{\rm{N}}}_{4}$ microresonator is $2.6\,{\rm{\mu }}{\rm{m}}$. Each resonance’s intrinsic linewidth ${\kappa }_{0}/2\pi$ and central frequency $\omega /2\pi$ are measured and fitted. The histogram of intrinsic quality factors ${Q}_{0}=\omega /{\kappa }_{0}$ of the $70,220$ resonances is shown in Fig. 2a. The most probable value is ${Q}_{0}=25\times {10}^{6}$, corresponding to a linear optical loss of $\alpha \approx 1.3\,{\rm{dB}}/{\rm{m}}$ (physical length). Figure 2c shows the measured ${Q}_{0}$ distribution over the entire wafer. Supplementary Materials Note 3 shows the wavelength-dependent ${\kappa }_{0}/2\pi$ over $1480$ to $1640\,{\rm{nm}}$ of one representative chip. Such a high $Q$ value is mandatory for coherent microcomb generation in the ${{\rm{Si}}}_{3}{{\rm{N}}}_{4}$ microresonator with $37.7\,{\rm{mW}}$ power in the bus waveguide.

There are two types of coherent microcombs, i.e. the bright dissipative Kerr soliton (DKS)^4,16–19^, and the dark pulse or platicon^20,42–45^. Figure 2b shows the measured microresonator’s integrated dispersion ${D}_{\mathrm{int}}$ (see definition in Materials and methods). Our ${{\rm{Si}}}_{3}{{\rm{N}}}_{4}$ microresonator features normal group velocity dispersion (GVD, ${D}_{2}\, <\, 0$), permitting platicon generation. In contrast, DKS requires anomalous GVD (${D}_{2}\, >\, 0$) that necessitates ${{\rm{Si}}}_{3}{{\rm{N}}}_{4}$ thickness above $600\,{\rm{nm}}$ and specific dispersion engineering^38,46^.

In addition, we emphasize another two advantages of using platicons instead of DKS for microwave generation. First, compared to DKS, platicons exhibit remarkably higher CW-to-pulse power conversion efficiency^47,48^. For $10.7\,{\rm{GHz}}$ repetition rate, we achieve $8 \%$ power conversion efficiency using platicons (discussed later), while the typical value is $\sim 0.2 \%$ for DKS^5,48^. Second, for DKS-based microwave generation, the CW pump must be filtered or blocked before photodetection of the pulse’s repetition rate^4–6^. Otherwise, the overwhelmingly strong pump can saturate the PD, yielding deteriorated power and phase noise of the microwave signal. Therefore, a filtering element, e.g. a notch filter, is required in the DKS-based microwave oscillator, which however complicates the overall system and photonic integration. In contrast, platicon-based microwave oscillator does not require such a filter, as in our case. In fact, the presence of the strong CW pump is beneficial. The pump beats against its two neighboring comb lines – with the highest power among all comb lines – and generates strong microwave signal at the repetition rate. In sum, we employ platicons for photonic microwave generation.

#### Photodetector

The high-speed PD is edge-coupled to the ${{\rm{Si}}}_{3}{{\rm{N}}}_{4}$ chip’s output waveguide, and is connected by a ground-signal-ground (GSG) probe for microwave signal output, as shown in Fig. 1d. The epitaxial structure of the PD chip is grown on a semi-insulating indium phosphide (InP) substrate^49^. Figure 1e shows the false-colored scanning electron microscope (SEM) image of the PD’s cross-section and multi-layer structure. The fabrication process of the PD chip starts with P-type contact metals (Ti/Pt/Au/Ti) deposition. Dry etching steps are then performed using inductively coupled plasma etching to form a triple-mesa structure. After the deposition of N-type contact metals (GeAu/Ni/Au), a benzocyclobutene (BCB) layer is implemented beneath the coplanar waveguides (CPWs). This approach eliminates the necessity for air-bridge structures, thus ensuring a consistent and stable connection between p-mesa and CPWs. Detailed fabrication process is found in Supplementary Materials Note 4.

The frequency response of the InGaAs/InP waveguide device is investigated by an optical heterodyne setup. Upon receiving a heterodyne beatnote between two tunable lasers with wavelengths near $1550\,{\rm{nm}}$, the PD outputs a high-frequency RF signal whose power is measured by a powermeter (Rohde & Schwarz NRP-Z58). Details of the characterization setup are found in ref. ^49^. A typical frequency response for 3 × 15 µm^2^ PD chip is shown in Fig. 2d. By a polynomial fit of the measured data, $3$-${\rm{dB}}$ bandwidth over $110\,{\rm{GHz}}$ is shown. The I-V characteristics of the 3 × 15 µm^2^ PD chip are measured by a semiconductor device analyzer. Figure 2e shows the measured dark current below $1\,{\rm{nA}}$, which out-performs commercial high-speed PDs. Figure 2f shows the responsivity as high as $0.3\,{\rm{A}}/{\rm{W}}$ and above $0.2\,{\rm{A}}/{\rm{W}}$ within $152\,{\rm{nm}}$ bandwidth. We note that, the current responsivity of our PD is mainly limited by the light coupling efficiency from the ${{\rm{Si}}}_{3}{{\rm{N}}}_{4}$ chip to the PD chip. An optimized spot-size converter on the PD’s input waveguide can increase the responsivity to $0.8\,{\rm{A}}/{\rm{W}}$ while maintaining the bandwidth. Figure 2g shows the voltage-dependent saturation property of the PD measured around $10\,{\rm{GHz}}$. The ideal relationship between RF power ${P}_{{\rm{RF}}}$ and DC photo-current *I*_dc_ is ${P}_{{\rm{RF}}}={I}_{{\rm{d}}{\rm{c}}}^{2}{R}_{{\rm{l}}{\rm{oad}}}/2$, assuming a 100% modulation depth (${I}_{{\rm{a}}{\rm{c}}}={I}_{{\rm{dc}}}$), where ${R}_{{\rm{l}}{\rm{oad}}}=50\ {{\Omega }}$ is the load resistance and ${I}_{{\rm{a}}{\rm{c}}}$ is AC photo-current.

### Narrow-linewidth laser and platicon microcomb

**Fig. 3:**
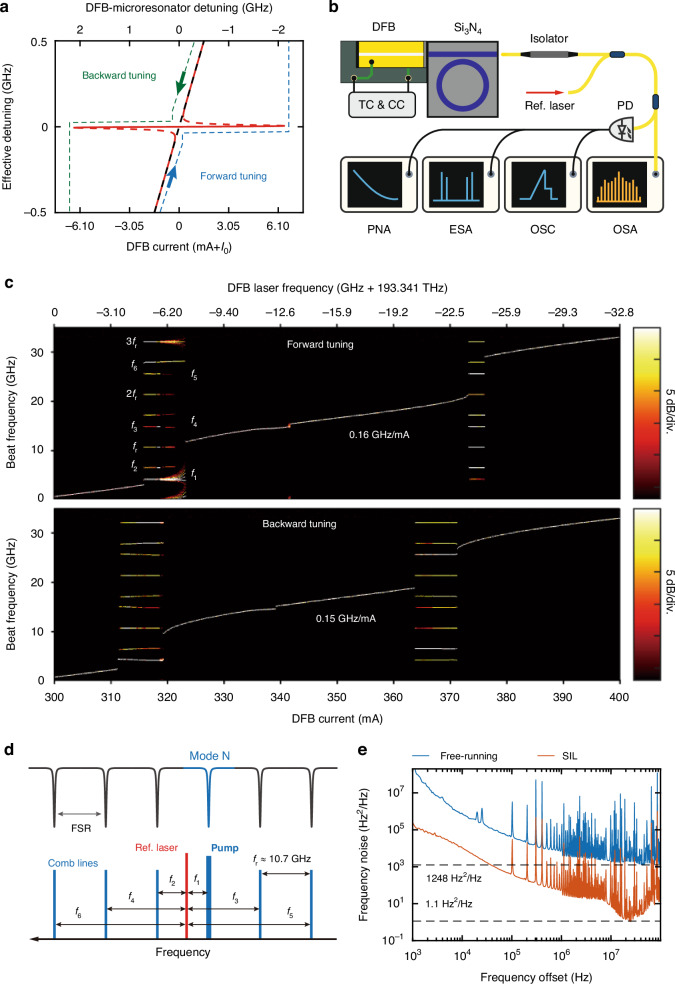
Characterization of laser self-injection locking. **a** Theoretical model of SIL dynamics using our experimental parameters. The red/gray line represents the steady-state solution with/without SIL. The vertical/top horizontal axis is the frequency detuning between the SIL/free-running DFB laser to the microresonator’s resonance. The bottom horizontal axis is the DFB current relative to ${I}_{0}$. The blue/green dashed line marks the forward/backward tuning curves. **b** Experimental setup. TC/CC, temperature/current control. OSC, oscilloscope. **c** Measured SIL dynamics. The vertical axis is the beat frequency $f$ between the reference laser and the SIL laser. In the forward/backward tuning, the DFB current increases/decreases and thus the laser frequency decreases/increases. Color bar marks the beat signal’s power. The flat segments signal the occurrence of SIL. The multiple beat frequencies (${f}_{1}$ to ${f}_{6}$ and ${f}_{{\rm{r}}}$ to ${3f}_{{\rm{r}}}$) signal platicon microcomb generation and are illustrated in **d**. **e** Frequency noise spectra of the free-running (blue) and SIL (red) DFB lasers. The frequency noise of the free-running/SIL DFB laser reaches ${1248/1.1\,{\rm{Hz}}}^{2}/{\rm{Hz}}$, corresponding to $7.8\,{\rm{kHz}}/6.9\,{\rm{Hz}}$ intrinsic linewidth

First, we characterize the laser dynamics and platicon states with the PD chip removed. The free-running DFB laser’s frequency is increased/decreased by decreasing/increasing the laser driving current. By scanning the DFB current in the forward/backward direction (increasing/decreasing the current) between $300$ and $400\,{\rm{mA}}$, the laser frequency is tuned by $32.8\,{\rm{GHz}}$, i.e. with tuning coefficient of $-0.328\,{\rm{GHz}}/{\rm{mA}}$. When the laser frequency approaches a microresonator resonance, light ${A}^{+}$ is coupled into the ${{\rm{Si}}}_{3}{{\rm{N}}}_{4}$ microresonator, as shown in Fig. 1a. Due to surface roughness and bulk inhomogeneity of the ${{\rm{Si}}}_{3}{{\rm{N}}}_{4}$ waveguide, back-scattered light ${A}^{-}$ is generated within the microresonator, and is injected back into the laser without an optical isolator. This back-scattered light can trigger laser self-injection locking (SIL)^50–53^, which locks the laser frequency $\nu$ to the microresonator resonance, as shown in Fig. 3a (see more description in Supplementary Materials Note 5). The locking range $\Delta {\nu }_{{\rm{lock}}}$ is defined as^51,52^

1 $$\Delta {\nu }_{{\rm{lock}}}\approx 2\nu \sqrt{1+{\alpha }_{{\rm{g}}}^{2}}\frac{\eta \beta }{{Q}_{{\rm{d}}}{R}_{0}}$$

where ${\alpha }_{{\rm{g}}}$ is the phase-amplitude factor of the DFB laser,$\eta ={\kappa }_{{\rm{ex}}}/({\kappa }_{{\rm{ex}}}{+\kappa }_{0})$ is the loading efficiency with ${\kappa }_{{\rm{ex}}}$ being the external coupling rate and ${\kappa }_{0}$ being the intrinsic loss, $\beta$ is the coupling rate between the counter-propagating modes (${A}^{+}$ and ${A}^{-}$) in the unit of $\kappa /2$ with $\kappa ={\kappa }_{{\rm{ex}}}{+\kappa }_{0}$, ${Q}_{{\rm{d}}}$ is the DFB laser cavity’s quality factor, ${R}_{0}$ is the reflectivity of the output facet of the DFB laser.

Once SIL occurs, the intrinsic laser linewidth is significantly suppressed due to the high quality factor ${Q}_{{\rm{m}}}=\omega /\kappa$ of the ${{\rm{Si}}}_{3}{{\rm{N}}}_{4}$ microresonator, which is much higher than ${Q}_{{\rm{d}}}$^50–52^. The suppression ratio of intrinsic laser linewidth can be expressed as

2 $$\frac{\delta {\nu }_{{\rm{l}}{\rm{ock}}}}{{\delta \nu }_{{\rm{free}}}}\approx \frac{{Q}_{{\rm{d}}}^{2}}{{Q}_{{\rm{m}}}^{2}}\frac{1}{64{\eta }^{2}{\beta }^{2}\left(1+{\alpha }_{{\rm{g}}}^{2}\right)}$$

where $\delta {\nu }_{{\rm{l}}{\rm{ock}}}$ (${\delta \nu }_{{\rm{free}}}$) is the locked (free-running) laser’s intrinsic linewidth. In addition, with sufficient intra-cavity optical power in the Kerr-nonlinear ${{\rm{Si}}}_{3}{{\rm{N}}}_{4}$ microresonator, nonlinear SIL can lead to platicon formation^36,54^.

The experimental setup to characterize nonlinear SIL dynamics and platicon generation is shown in Fig. 3b. A tunable diode laser (Toptica CTL 1550) is frequency-stabilized to a self-referenced fiber optical frequency comb (Quantum CTek). It serves as a reference laser to beat against the DFB laser emitted from the ${{\rm{Si}}}_{3}{{\rm{N}}}_{4}$ chip. The beat signal’s frequency $f$ is recorded by an electronic spectrum analyser (ESA, Rohde & Schwarz FSW43), and the optical spectrum is recorded by an optical spectrum analyser (OSA, Yokogawa AQ6370D).

As shown in Fig. 3c, while the beat frequency $f$ has a global monotonic dependence on the DFB current, it also exhibits multiple flat segments that stay nearly constant when the DFB current varies by less than $10\,{\rm{mA}}$. For example, in the backward tuning, $f$ is nearly constant from $\sim\!\! 372$ to $\sim \!\!363\,{\rm{mA}}$ and from $\sim \!\!320$ to $\sim\!\! 311\,{\rm{mA}}$. Such behaviors signal the occurrence of SIL and platicon generation. Degrading SIL performance can result from the presence of the ${{\rm{Si}}}_{3}{{\rm{N}}}_{4}$ bus waveguide that serves as an extra Fabry-Perot cavity with weak reflection (see Supplementary Materials Note 6).

The multiple beat frequencies (${f}_{1}$ to ${f}_{6}$) in Fig. 3c within the SIL region, e.g. around $320\,{\rm{mA}}$ DFB current in the forward tuning, are illustrated in Fig. 3d. Assuming that the pump laser frequency is close to the resonance mode $N$. Then ${f}_{1}$ to ${f}_{6}$ are the beat frequencies between the reference laser and the platicon’s comb lines including the pump. Meanwhile, $f=m{\rm{\cdot }}{f}_{{\rm{r}}}$ ($m$ is an integer) is the beat frequencies among the comb lines, where ${f}_{{\rm{r}}}\approx 10.7\,{\rm{GHz}}$ is the mode spacing (i.e. the platicon’s repetition rate). Note that, our ESA has $43.5\,{\rm{GHz}}$ measurement bandwidth, allowing detection of microwave tones up to the fourth harmonics ${4f}_{{\rm{r}}}$ of $42.8\,{\rm{GHz}}$. As the DFB current is tuned to $372\,{\rm{mA}}$ in the forward tuning, the laser frequency approaches the $N+1$ resonance mode, leading to another SIL region. Identical behaviors are found in the backward tuning. Considering both directions, the DFB current can be tuned by $12.3\,{\rm{mA}}$ in the SIL region, corresponding to a full locking range about $4.0\,{\rm{GHz}}$. Such nonlinear SIL dynamics with platicon generation are similar to the dynamics with DKS generation in ref. ^53^. In addition, we also observe in Fig. 3c that, in the forward tuning with DFB current near $320\,{\rm{mA}}$, the ${f}_{1}$ beat signal becomes noisy. We experimentally identify that, with DFB current near 320 mA, the platicon experiences transition to a breathing platicon crystal^55^. In the breathing state, the dark pulse’s repetition rate is perturbed. Therefore, in the following experiments, we operate system away from this special regime.

In the SIL region and with $316.61\,{\rm{mA}}$ current, the output microcomb power excluding the pump line from the ${{\rm{Si}}}_{3}{{\rm{N}}}_{4}$ chip is about $1.1{\rm{mW}}$. When the DFB laser frequency is off-resonant, the CW output power from the ${{\rm{Si}}}_{3}{{\rm{N}}}_{4}$ chip is $14\,{\rm{mW}}$. Therefore, the CW-to-platicon conversion efficiency is calculated as $8 \%$. Figure 3e shows the optical frequency noise of the SIL laser characterized by a delayed self-heterodyne method^56^ (see Supplementary Materials Note 7). The lowest frequency noise is $1.1\,{{\rm{Hz}}}^{2}/{\rm{Hz}}$, corresponding to $6.9\,{\rm{Hz}}$ intrinsic linewidth and $31$-${\rm{dB}}$ noise suppression on the free-running DFB laser.

### Microwave generation and noise quenching

**Fig. 4:**
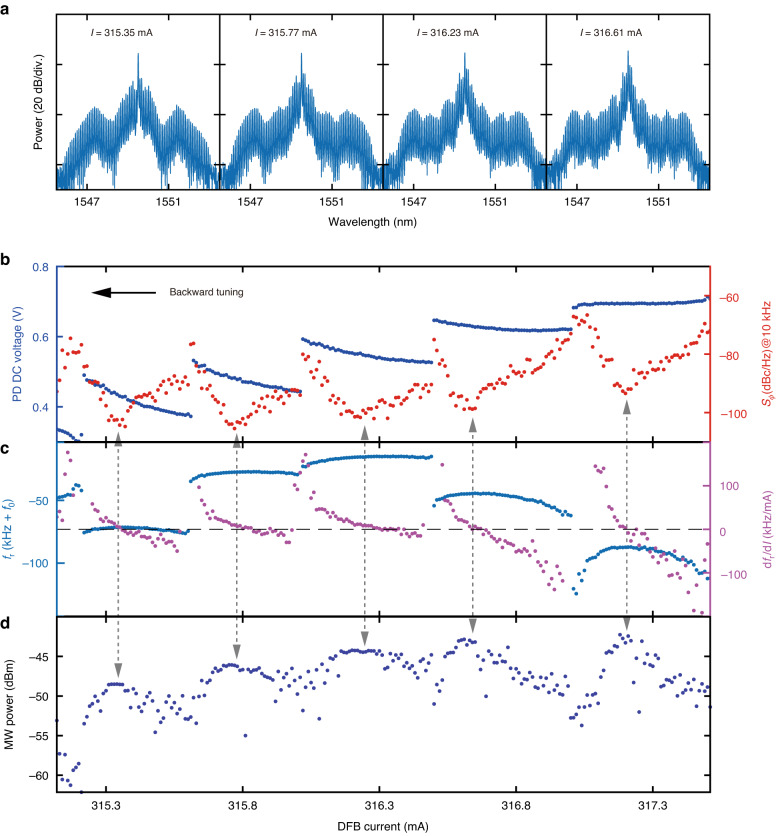
Observation and characterization of noise-quenching dynamics within discrete platicon states. **a** Optical spectra of four platicon states with DFB current values of $313.25,315.77,316.23$ and $316.61\,{\rm{mA}}$. **b** Measured platicon’s optical power (proportional to the PD’s DC output voltage, blue dots) and the microwave phase noise ${S}_{\phi }$ at $10\,{\rm{kHz}}$ Fourier offset frequency of the ${f}_{{\rm{r}}}\approx 10.7\,{\rm{GHz}}$ carrier (red dots). **c** Measured microwave carrier frequency of ${f}_{{\rm{r}}}\approx 10.7\,{\rm{GHz}}$ (light blue dots) and the derivative of ${f}_{{\rm{r}}}$ to the DFB current (i.e. ${\rm{d}}{f}_{{\rm{r}}}/{\rm{d}}I$, purple dots). ${f}_{0}=10.685550\,{\rm{GHz}}$ is a frequency offset. **d** Measured microwave power of the ${f}_{{\rm{r}}}\approx 10.7\,{\rm{GHz}}$ carrier. Within each platicon state, the gray dashed arrows across **b–d** highlight the noise-quenching states, where the local minima of ${S}_{\phi }$ always coincide with the local maxima of ${f}_{{\rm{r}}}$ and microwave power, as well as ${\rm{d}}{f}_{{\rm{r}}}/{\rm{d}}I=0$ points

The demonstrated narrow linewidth and ultralow noise of the SIL laser are critical for low-noise microwave generation. Upon photodetection of the platicon pulse stream, the PD outputs microwaves of carrier frequency corresponding to the platicon’s repetition rate of ${f}_{{\rm{r}}}\approx 10.7\,{\rm{GHz}}$ and harmonics of ${f}_{{\rm{r}}}$. First, we focus on the ${f}_{{\rm{r}}}\approx 10.7\,{\rm{GHz}}$ microwave and study the noise transduction scheme. Figure 4a shows four different platicon states accessed in the backward tuning. We continuously tune the DFB current, and simultaneously monitor the platicon’s optical power (proportional to the PD’s DC output voltage, Fig. 4b) and ${f}_{{\rm{r}}}$ (Fig. 4c). For the microwave carrier at ${f}_{{\rm{r}}}$, we also monitor the microwave power (Fig. 4d) and the phase noise ${S}_{\phi }$ at $10\,{\rm{kHz}}$ Fourier offset frequency (Fig. 4b). These results are displayed and compared in Fig. 4b–d, where two intriguing phenomena are observed. First, the switching dynamics among different platicon states endows discrete step features in the traces of PD’s DC voltage (Fig. 4b) and ${f}_{{\rm{r}}}$ (Fig. 4c). Second, as shown in Fig. 4b, within each platicon state, a local minimum of ${S}_{\phi }$ is found, providing additional noise quenching. Interestingly yet comprehensibly, such local minima of ${S}_{\phi }$ always coincide with local maxima of ${f}_{{\rm{r}}}$, where ${f}_{{\rm{r}}}$ has null dependence to the DFB current $I$, i.e. ${\rm{d}}{f}_{{\rm{r}}}/{\rm{d}}I=0$ as marked in Fig. 4c purple data. It means that ${f}_{{\rm{r}}}$ is insensitive to current fluctuation and noise. Since the spectral purity is improved with minimized ${S}_{\phi }$, the local maxima of microwave power always coincide with local minima of ${S}_{\phi }$, as shown in Fig. 4d. Gray dashed arrows highlight the coincidence of local minima of ${S}_{\phi }$, local maxima of ${f}_{{\rm{r}}}$ and microwave power, and ${\rm{d}}{f}_{{\rm{r}}}/{\rm{d}}I=0$ points. The platicon states corresponding to four local minima of ${S}_{\phi }$ are shown in Fig. 4a.

**Fig. 5:**
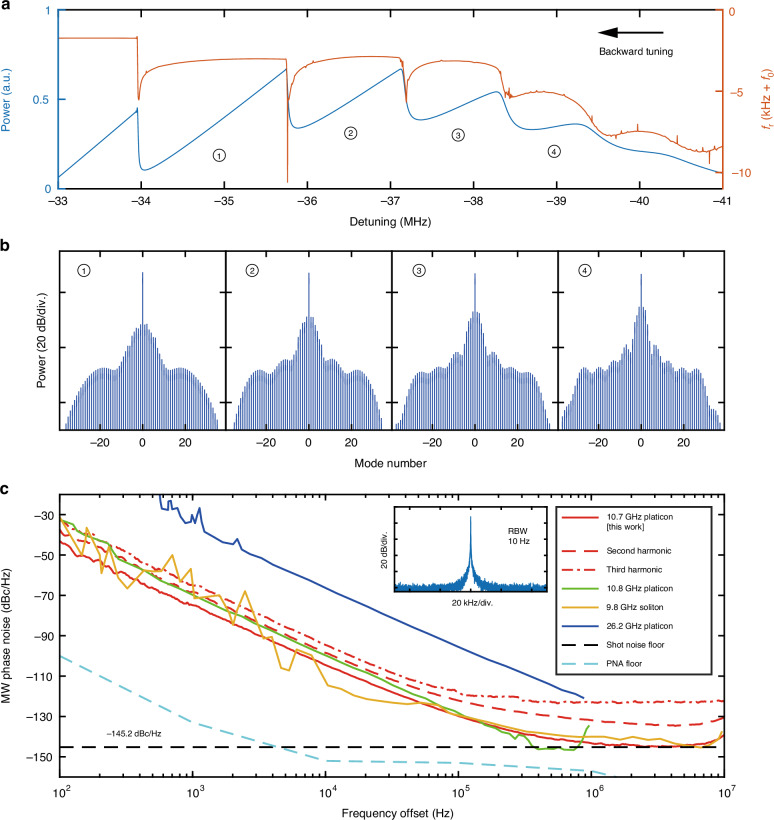
Simulated platicon switching dynamics and measured microwave phase noise spectra. **a** LLE simulation of platicon’s optical power (blue curve) and repetition rate ${f}_{{\rm{r}}}$ (red curve) with increasing DFB laser frequency (black arrow). Discrete steps corresponding to different platicon states are numerically marked. **b** Simulated platicon spectra corresponding to the marked states in **a**. **c** Phase noise spectra of our $10.7\,{\rm{GHz}}$ microwave (solid red line), the second harmonic ($21.4\,{\rm{GHz}}$, dashed red line), and the third harmonic ($32.1\,{\rm{GHz}}$, dashdot red line), in comparison with the spectra in ref. ^54^ (green line), ref. ^5^ (orange line), and ref. ^36^ (blue line), as well as the PNA floor (light dashed blue line) and shot noise floor (dashed black line). While we use an integrated PD chip, refs. ^5,36,54^ use commercial, non-integrated PD via fiber connection. Inset is the power spectrum of the $10.7\,{\rm{GHz}}$ microwave in our work. RBW, resolution bandwidth

To validate our experimental observation, we further numerically model the nonlinear system using Lugiato-Lever Equation (LLE)^57^. The platicon state can be generated by scanning the laser frequency from the blue detuning to the red detuning of the frequency-shifted pumped mode ($\mu =$0)^42^. The pump mode’s frequency shift is caused by SIL, equivalent to the effect of avoided mode crossing (AMX) in the microresonator^20^. The FSR of the DFB laser’s cavity is measured as $29\,{\rm{GHz}}$, which is nearly $3$-times larger than the ${{\rm{Si}}}_{3}{{\rm{N}}}_{4}$ microresonator FSR. Considering relatively wide linewidth of the laser cavity, the microresonator modes such as $\mu =\pm 3,\,\pm 6$ can also couple to the laser mode and thus be frequency-shifted. Using our experimental parameters, discrete platicon states and local maxima of ${f}_{{\rm{r}}}$ are reproduced in simulation by backward tuning, as shown in Fig. 5a. More details are found in Supplementary Materials Note 8. The varying ${f}_{{\rm{r}}}$ results from mode-coupling-induced dispersive waves^58,59^. It is worth to note that the detuning in Fig. 5a corresponds to the effective detuning in Fig. 3a since the DFB laser frequency is tightly locked on the ${{\rm{Si}}}_{3}{{\rm{N}}}_{4}$ microresonator. In addition, note that Fig. 4b shows decreasing microresonator output power with decreasing DFB current, while Fig. 5a shows increasing microresonator intracavity power with decreasing DFB current. This is due to that the microresonator’s output power and its intracavity power have opposite trends with the DFB current. The simulated optical spectra of different platicon states are shown in Fig. 5b, resembling the experimental spectra. The simulated time-domain pulse waveforms are found in Supplementary Materials Note 8. More comprehensive theoretical and numerical models are found in our parallel study^60^.

Finally, we couple the PD chip to the ${{\rm{Si}}}_{3}{{\rm{N}}}_{4}$ chip, enabling photonic microwave generation up to $110\,{\rm{GHz}}$ within the PD’s bandwidth. We set $315.35\,{\rm{mA}}$ DFB current such that the system is operated in the most effective noise-quenching state as shown in Fig. 4. We analyze the $10.7\,{\rm{GHz}}$ microwave and its second ($21.4\,{\rm{GHz}}$ in the K-band) and third harmonics ($32.1\,{\rm{GHz}}$ in the ${{\rm{K}}}_{{\rm{a}}}$-band). The RF power exported from the PD chip is $-28/\!-\!30/\!-\!33\,{\rm{dBm}}$ for the $10.7/21.4/32.1\, {\rm{GHz}}$ microwave. The microwave’s phase noise is measured with $20$-times averaging by a phase noise analyzer (PNA, Rohde & Schwarz FSWP50), as shown in Fig. 5c. The $10.7\,{\rm{GHz}}$ microwave’s phase noise reaches $-75/\!\!-105/\!\!-130\,{\rm{dBc}}/{\rm{Hz}}$ at $1/10/100\,{\rm{kHz}}$ Fourier offset frequency, and its power spectrum with $6.3\,{\rm{mHz}}$ linewidth is shown in Fig. 5c inset. The phase noise of the second-/third-harmonic microwave is about $6.0/9.5\,{\rm{dB}}$ higher, in agreement with the scaling law of $20\log N$ ($N=2$ or $3$). More phase-noise experiments with different DFB currents, as well as with another ${{\rm{Si}}}_{3}{{\rm{N}}}_{4}$ microresonator and another DFB laser, are presented in our parallel study^60^. In comparison, we plot the results from refs. ^5,36,54^ which use commercial, non-integrated PDs via fiber connection. The measured Allan deviations of the SIL laser frequency and the microwave frequency are found in Supplementary Materials Note 7. The resemblance in trend indicates that the long-term stability of the microwave is dominated by the SIL laser frequency. We further compare the phase noise of our microcomb-based photonic microwave oscillator with those of commercial microwave oscillators in Supplementary Materials Note 9, where our photonic microwave oscillator benefits a balanced feature on low phase performance and small size.

## Discussion

In conclusion, we have demonstrated a fully hybrid-integrated photonic microwave oscillator at $10.7\,{\rm{GHz}}$ using a platicon microcomb. Such a hybrid chip device, occupying a footprint of only $76\,{{\rm{mm}}}^{2}$, consists of a high-power DFB laser, a high-$Q$ ${{\rm{Si}}}_{3}{{\rm{N}}}_{4}$ microresonator, and a high-speed PD. Each component represents the state of the art of its own class, yet can be manufactured in large volume with low cost using established CMOS and III-V foundries of integrated photonics.

Leveraging laser self-injection locking, the laser frequency noise is suppressed by $31\,{\rm{dB}}$, yielding an intrinsic linewidth of $6.9\,{\rm{Hz}}$ and platicon microcomb generation. Harnessing the noise-quenching dynamics within discrete platicon states, the PD chip further outputs a $10.7\,{\rm{GHz}}$ low-noise microwave carrier of $6.3\,{\rm{mHz}}$ linewidth, and harmonics of $10.7\,{\rm{GHz}}$ benefiting from the PD’s $110\,{\rm{GHz}}$ bandwidth. In the future, the long-term stability of the three-in-one **–** hybrid laser, comb and microwave **–** can be improved by PDH-locking to an ultra-stable cavity^61,62^. Recent advances^63–66^ in heterogeneous integration of III-V, ${{\rm{Si}}}_{3}{{\rm{N}}}_{4}$ and modulators open a path towards chip devices of further reduced sizes, improved stability and added frequency tunability. Up-shifting the microwave frequency, even into the millimeter-wave band, can be simply realized with ${{\rm{Si}}}_{3}{{\rm{N}}}_{4}$ microresonators of larger FSR^67,68^ and modified uni-traveling-carrier (MUTC) InP PD chips^49^. Such low-noise microwaves from hybrid photonic chips of small size, weight and power consumption can reinvigorate our information technology and applications for microwave photonics, terrestrial broadband, traffic control, tracking, analog-to-digital conversion, wireless networks, space links, and electron paramagnetic resonance spectroscopy.

## Materials and methods

### Characterization of microresonator dispersion

We use a vector spectrum analyser (VSA)^41^ to measure the central frequency $\omega /2\pi$ and loaded linewidth $\kappa /2\pi =({\kappa }_{0}+{\kappa }_{{\rm{e}}{\rm{x}}})/2\pi$ of each resonance within $1480$ to $1640\,{\rm{nm}}$. The intrinsic loss ${\kappa }_{0}$ and external coupling rate ${\kappa }_{{\rm{e}}{\rm{x}}}$ can be fitted based on the resonance’s transmission profile^69,70^, extinction ratio^71^ and phase response^41^. The intrinsic (${Q}_{0}$) and loaded (${Q}_{{\rm{m}}}$) quality factors of each resonance can be calculated as ${Q}_{0}=\omega /{\kappa }_{0}$ and ${Q}_{{\rm{m}}}=\omega /\kappa$. Due to the presence of microresonator dispersion, the measured resonance grid deviates from an equidistant grid. Thus the ${\mu }^{{\rm{th}}}$ resonance frequency *ω*_*μ*_ can be written as

3 $${\omega }_{\mu }={\omega }_{0}+{D}_{1}\mu +\frac{{D}_{2}{\mu }^{2}}{2}+\frac{{D}_{3}{\mu }^{3}}{6}+\,\ldots$$

4 $$={\omega }_{0}+{D}_{1}\mu +{D}_{{\rm{i}}{\rm{nt}}}$$

where ${\omega }_{0}$ is the reference resonance frequency, ${D}_{1}/2\pi$ is the microresonator FSR, ${D}_{2}$ describes the GVD, ${D}_{3}$ is higher-order dispersion term. With known ${\omega }_{\mu }$, ${\omega }_{0}$ and fitted ${D}_{1}$, the microresonator’s integrated dispersion ${D}_{\mathrm{int}}={\omega }_{\mu }-{\omega }_{0}-{D}_{1}\mu$ can be plotted.

## Supplementary information

Supplementary Information

## Data Availability

The code and data used to produce the plots within this work are available in Zenodo (10.5281/zenodo.14944989).
