# Supplementary material for: A chip-integrated comb-based microwave oscillator
Source: Light Sci Appl. 2025 Apr 30;14:179. doi: 10.1038/s41377-025-01795-0 (PMC12041566; doi:10.1038/s41377-025-01795-0)
Supplement: Supplementary file 1 — Supplementary Information [file 41377_2025_1795_MOESM1_ESM.pdf]

Supplementary Information for A chip-integrated comb-based microwave oscillator

Wei Sun,[1,*] Zhiyang Chen,[1,*] Linze Li,[2,*] Chen Shen,[1,3,*] Kunpeng Yu,[1,4] Shichang Li,[1] Jinbao Long,[1] Huamin Zheng,[1] Luyu Wang,[2] Tianyu Long,[2] Qiushi Chen,[2] Zhouze Zhang,[2] Baoqi Shi,[1] Lan Gao,[1] Yi-Han Luo,[1] Baile Chen,[2,†] and Junqiu Liu[1,4,‡]

[1]*International Quantum Academy, Shenzhen 518048, China*
[2]*School of Information Science and Technology, ShanghaiTech University, Shanghai 201210, China*
[3]*Qaleido Photonics, Shenzhen 518048, China*
[4]*Hefei National Laboratory, University of Science and Technology of China, Hefei 230088, China*

Supplementary Note 1. DFB laser characterization

The performance of the DFB laser is characterized as shown in Fig. S1. The DFB can emit 160 mW laser at 500 mA current with the laser threshold of 55 mA. In the 500 mA current range, the DFB laser's emission wavelength can be tuned over 1 nm from the starting wavelength 1548.786 nm at the temperature of 30°C. In the edge-coupling scheme, the coupling efficiency is measured at different currents from 280 mA to 480 mA, with a mean coupling efficiency of $\sim 23\%$.

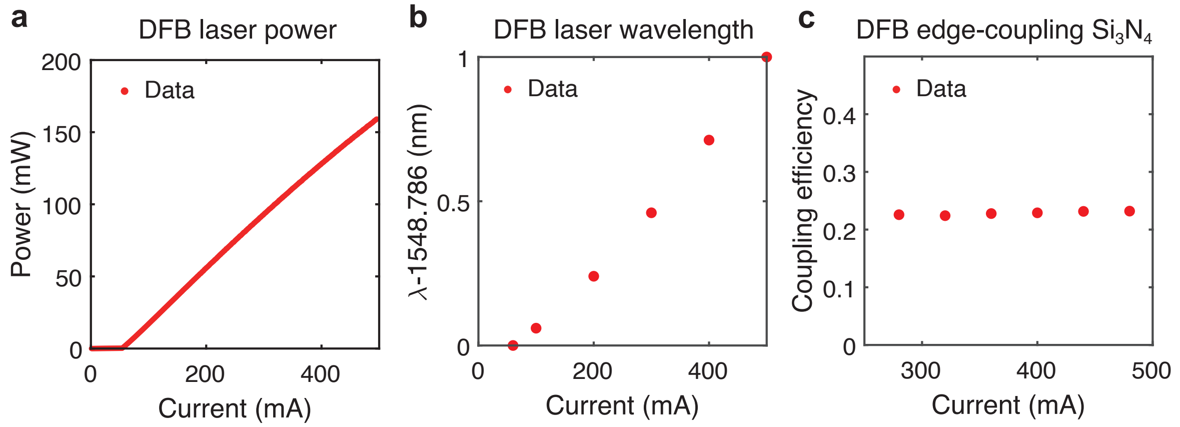

Figure S1: DFB laser characterization. **a**. Measured DFB output power versus the applied current. **b**. Measured DFB laser frequency versus the applied current. **c**. Measured coupling efficiency between the DFB laser chip and the Si_3N_4 chip.

Supplementary Note 2. Fabrication process of Si_3N_4 chips

The high-Q Si_3N_4 microresonator chips are fabricated using an optimized DUV subtractive process on 6-inch wafers[1]. The process flow is shown in Fig. S2. First, an LPCVD Si_3N_4 film is deposited on a clean thermal wet SiO_2 substrate. Compared to thick Si_3N_4 films suffering from cracks due to intrinsic tensile stress, the 300-nm-thick Si_3N_4 films do not exhibit any cracks during the fabrication. A SiO_2 film is further deposited as an etch hardmask. Afterwards, DUV stepper photolithography is performed, followed by dry etching to transfer the pattern from the photoresist to the SiO_2 hardmask and then to the Si_3N_4 layer. In the dry etching, etchants of CHF_3 and O_2 are used to create ultra-smooth and vertical etched surfaces, which are critical for low optical loss in waveguides. Then the photoresist is removed and a thermal annealing is applied in the nitrogen atmosphere at 1200°C to drive out hydrogen contents that cause optical absorption loss in the waveguides. Then 3-μm-thick SiO_2 top cladding is deposited on the wafer and thermally annealed again at 1200°C to drive out hydrogen contents. Afterwards, platinum heaters are deposited on the substrate via an electron-beam evaporator, and patterned via a lift-off process. The metal heaters have negligible impact on the optical loss of the Si_3N_4 waveguide due to the thick SiO_2 cladding. Contact UV photolithography and additional deep dry etching are performed to create smooth chip facets and define the chip size, critical for later hybrid integration and packaging. Finally, the wafer is separated into individual chips by backside grinding.

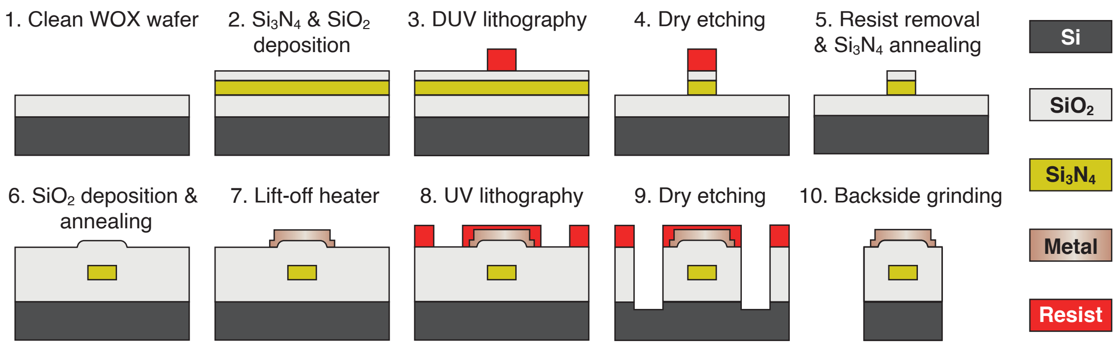

Figure S2: The DUV subtractive process flow of the 6-inch-wafer Si_3N_4 foundry fabrication. WOX, thermal wet oxide (SiO_2).

Supplementary Note 3. Loss measurement of a microresonator over a wide bandwidth

By fitting the transmission spectrum of a resonance mode, the intrinsic loss $\kappa_0/2\pi$ and the external coupling rate $\kappa_{ex}/2\pi$ can be extracted, as shown in Fig. S3a. The resonance mode locates at frequency $\nu = \omega/2\pi = 200.5$ THz. The intrinsic loss is $\kappa_0/2\pi = 8.6$ MHz, and the external coupling rate is $\kappa_{ex}/2\pi = 4.7$ MHz, which indicates an under coupling of the resonance. Scanning a laser from $\nu = 202.6$ THz to 182.8 THz (wavelength from 1480 to 1640 nm), the loss over a large bandwidth can be traced, as shown in Fig. S3b. The overall measurements are performed using a vector spectrum analyser[2].

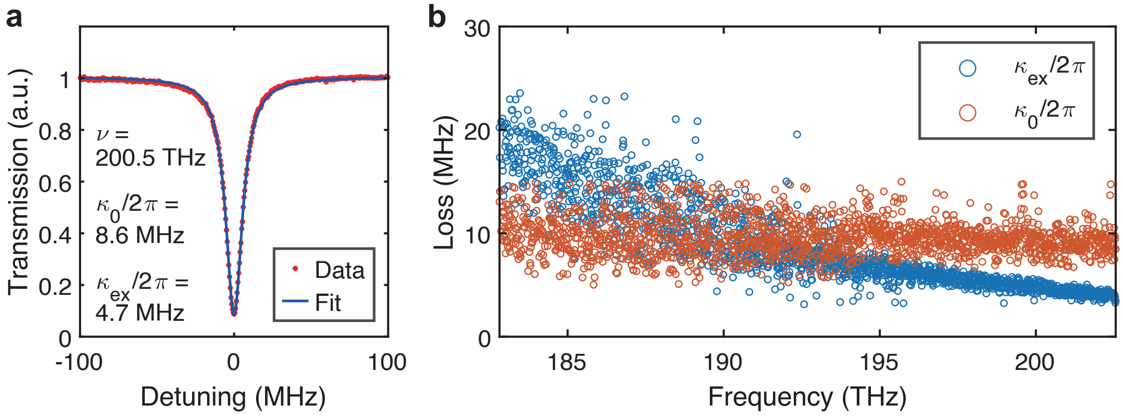

Figure S3: Intrinsic and external coupling loss of the Si_3N_4 microresonator. a. Resonance fitting at frequency $\nu = 200.5$ THz. Red dots are measured data. Blue line is the corresponding fit. **b**. Loss measurement over 20 THz. The red circles are data of intrinsic loss. The blue circles are data of external coupling loss.

Supplementary Note 4. Fabrication process of photodetector chips

The epitaxial structure of the PD chip is grown by metal-organic chemical vapor deposition (MOCVD) on a 2-inch semi-insulating InP wafer. The fabrication process is shown in Fig. S4. The fabrication process begins with the metal deposition of the p-contacts (step 2), composed of Ti/Pt/Au layers (20/30/120 nm). Then, a tipple-mesa structure is defined by a combination of dry and wet etch process (step 3). Inductively coupled plasma (ICP) dry etching is performed to achieve vertical sidewalls and precise control of the first p-mesa and the second waveguide mesa. The third n-mesa is terminated at the semi-insulating InP substrate by wet chemical etching (H_3PO_4:HCl = 1:3) to ensure electrical isolation. After the deposition of GeAu/Ni/Au layers (40/20/120 nm) as the n-contact metals (step 4), rapid thermal annealing process at 360°C is conducted for lower contact resistance. A benzocyclobutene (BCB) layer is implemented to passivate the sidewall of p-mesa and provide stable support for the subsequent electrodes (step 5). Finally, the p-mesa is connected to coplanar waveguide (CPWs) pads of 50 Ω characteristic impedance by metal deposition (step 6). Such a non-suspended structure eliminates the necessity for air-bridge structures, consequently ensuring a consistent and stable connection between p-mesa and CPWs.

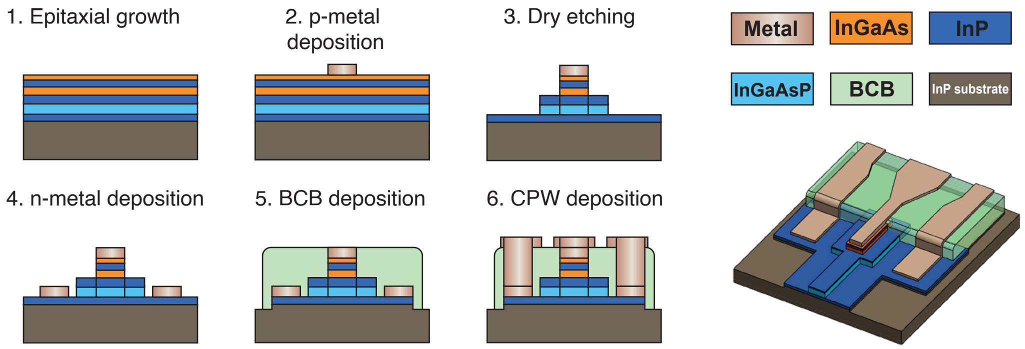

Figure S4: Fabrication process of the photodetector chip. BCB, benzocyclobutene. CPW, coplanar waveguide.

Supplementary Note 5. Self-injection locking to the microresonator

A comprehensive theory on self-injection locking of a laser diode to a microresonator is presented in Ref.[3,4]. By a simple model and reasonable approximation, the stable solution of the combined system is described as

$$\xi = \zeta + \frac{K}{2}\frac{2\zeta\cos\bar{\psi} + \left(1+\beta^2-\zeta^2\right)\sin\bar{\psi}}{\left(1+\beta^2-\zeta^2\right)^2+4\zeta^2}, \tag{S1}$$

where ξ represents the frequency detuning between the generated light of the system and the resonance of the microresonator, ζ represents the frequency detuning between the free-running laser and the resonance of the microresonator, K denotes the combined coupling coefficient of the system, $\bar{\psi} = \psi_0 + \kappa\tau_{\mathrm{s}}\zeta/2$ is the phase delay between the laser and the microresonator, and β is the back-scattering-related mode splitting of the microresonator. Parameters ξ, ζ and β are in the unit of $\kappa/2$ where $\kappa/2\pi$ is the loaded linewidth of the Si_3N_4 microresonator.

Experimentally, κ is measured as $2\pi \times 17.6$ MHz. The intrinsic loss rate κ_0 and the external coupling rate κ_{ext} are $2\pi \times 11.9$ MHz and $2\pi \times 6.7$ MHz, respectively. The time delay τ_{s} between the DFB laser and the microresonator is estimated as 3×10^{-11} s. The mode splitting of the resonance β is nearly negligible for high-Q microresonator. In case of $\kappa\tau_{\mathrm{s}} \ll 1$, $\beta \ll 1$, which is true in our experiment, and $\psi_0 = 0$ for convenience, Eq. S1 can be re-written as

$$\xi = \zeta + K\frac{\zeta}{\left(1+\zeta^2\right)^2}. \tag{S2}$$

For sufficiently large K ($K \gg 4$), the locking range in the SIL region is $\Delta\omega \approx 3\sqrt{3}K\kappa/16 \approx 2\omega\sqrt{1+\alpha_{\mathrm{g}}^2}\eta\beta/(Q_{\mathrm{d}}R_{\mathrm{o}})$ by solving $\partial\xi/\partial\zeta = 0$. As measured in the main text, the locking range is about $\Delta\omega/2\pi \approx 4$ GHz, and K is estimated as 700. β is set to 0.004 for calculation. Applying the above parameters in Eq. S1, the solution is shown in Fig. 3a in the main text.

Supplementary Note 6. Self-injection locking to the waveguide Fabry-Pérot cavity

The DFB laser's frequency can be locked to the Si_3N_4 bus waveguide with edge-coupling to the microresonator. In this case, the bus waveguide behaves as a Fabry-Pérot (FP) cavity. The locked laser frequency is measured by the beat-note frequency with the reference frequency. The length of the DFB and the Si_3N_4 FP cavity are 1.5 mm and 5.0 mm, respectively. The forward and backward dynamics of laser frequency tuning are presented in Fig. **S5**b.

The quarter-wave shifted DFB laser is simulated by the transmission matrix theory of gratings. The effective refractive index of the active grating is set to $n_{\mathrm{e}} = 3.3$ with grating couple efficiency $\kappa_{\mathrm{g}} = 10.3$ cm^{-1}. The effective refractive index of the Si_3N_4 waveguide is set to $n_{\mathrm{p}} = 1.9$. The laser frequency is linearly dependent on the DFB current in free-running state, as the green lines in Fig. **S5**b. The effective reflectivity for the hybrid system is defined by[5]

$$r_{\mathrm{eff}} = r_{\mathrm{g}}(n_{\mathrm{e}},\kappa_{\mathrm{g}}) + \frac{t_{\mathrm{g}}^2(n_{\mathrm{e}},\kappa_{\mathrm{g}})\cdot r_{\mathrm{p}}e^{-2j\beta_{\mathrm{p}}L_{\mathrm{p}}}}{1+r_{\mathrm{g}}(n_{\mathrm{e}},\kappa_{\mathrm{g}})\cdot r_{\mathrm{p}}e^{-2j\beta_{\mathrm{p}}L_{\mathrm{p}}}}, \tag{S3}$$

where r_{g} and t_{g} are the effective reflection and transmission coefficients for the front DFB grating, respectively. And r_{p} is the reflection coefficient for the interface of the Si_3N_4 waveguide, $\beta_{\mathrm{p}} = n_{\mathrm{p}}k$ where k is the wave vector. L_{p} is the length of the Si_3N_4 waveguide. The round-trip light field in the cavity is

$$E_{\mathrm{rp}} = r_{\mathrm{r}}(n_{\mathrm{e}},\kappa_{\mathrm{g}})\cdot r_{\mathrm{eff}}e^{-2j\beta_{\mathrm{a}}L_{\mathrm{a}}}, \tag{S4}$$

where r_{r} is the effective reflection coefficient for the rear DFB grating, $\beta_{\mathrm{a}} = n_{\mathrm{e}}k$, and L_{a} is the length of DFB. The laser frequency is obtained by the cavity round-trip phase condition. Through forward and backward current tuning, the laser frequency dynamics is presented as blue and red lines in Fig. **S5**b. The FSR of the Si_3N_4 FP cavity about 15.9 GHz indicates that the laser mode hopping is due to the coupling of the DFB mode and the FP mode. The hysteresis loop can change with different phases between the DFB laser and the Si_3N_4 waveguide. The simulation with 0.1π phase is shown in Fig. **S5**b lower panel.

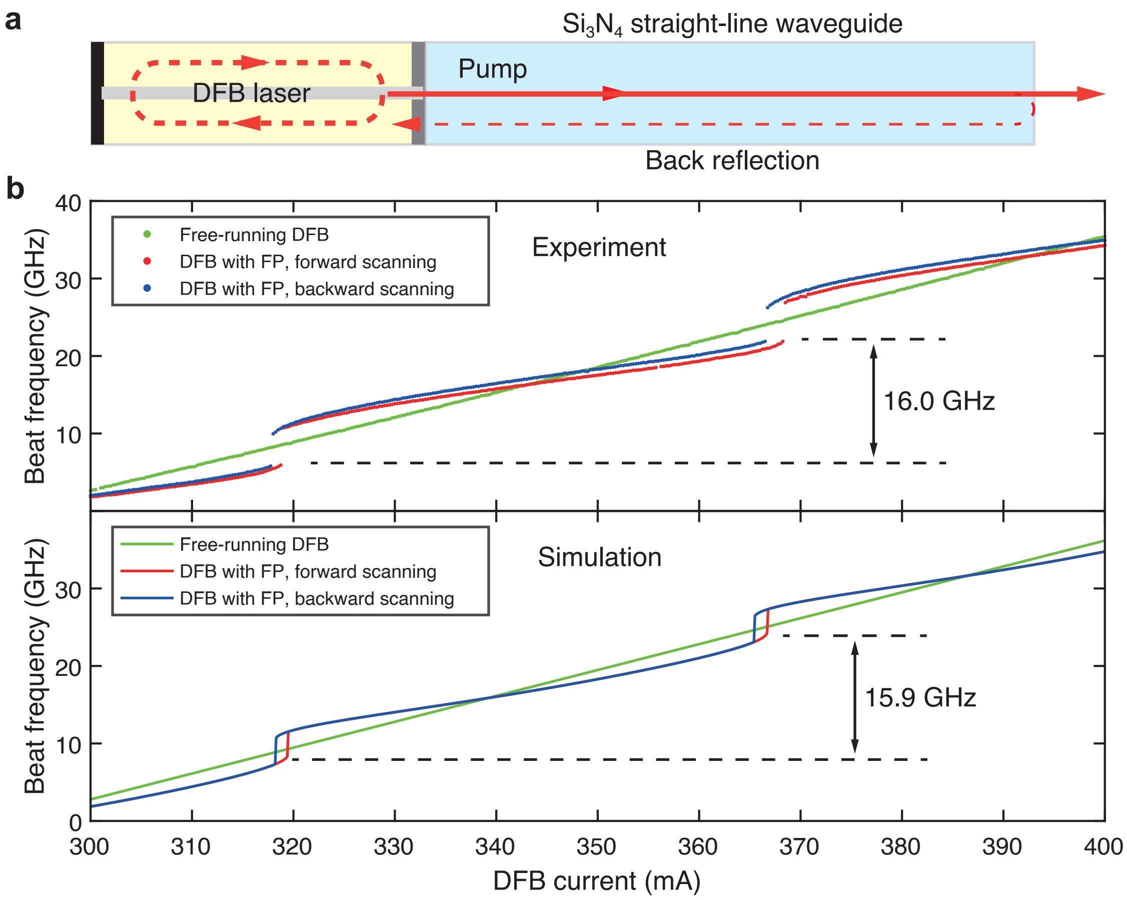

Figure S5: Laser self-injection locking to the Si_3N_4 bus waveguide. **a**. Schematic of the DFB laser locked to an FP cavity. **b**. Hysteresis behavior when scanning the DFB laser frequency via the DFB current. When the current is tuned from 300 to 400 mA, the frequency mode hopping is observed at the FP cavity's resonant frequency (red curve). While in the backward tuning, the mode hopping is also observed despite that the hopping current point is not the same as that in the forward tuning. The blue curve shows a slight current offset. The green line is the frequency tuning of the free-running DFB. The simulation is presented in the lower panel using the transmission matrix method and coupled-mode theory. The mode hopping period of about 15.9 GHz (experiment about 16.0 GHz) corresponds to the FSR of the Si_3N_4 FP cavity.

Supplementary Note 7. Frequency noise and Allan deviation measurement

The laser frequency noise is measured by a delayed self-heterodyne interferometer (DSHI)[6]. The experimental setup is shown in Fig. S6a. The laser passes a polarization controller (PC) with its polarization aligned to the slow axis of the polarization-maintained fiber, which ensures the largest diffraction efficiency of the followed acousto-optic modulator (AOM). The modulation frequency on the AOM is 80 MHz. Parallel to the path of the light through the AOM, the other branch is sent into a 2-km-long fiber. The time delay τ_{d} between the two branches is estimated far less than the coherent time τ_{c} of the laser, i.e. $\tau_{\mathrm{d}} \ll \tau_{\mathrm{c}}$, to ensure a sub-coherent measurement. A fiber coupler combines the lights in both branches, with a PC in one path to ensure the same polarization in the fiber coupler. The beat signal of the two branches is detected by a photodetector (PD, Finisar XPDV3120R). The power spectral density (PSD) of the phase noise $S_{\Delta\phi}(f)$ is measured by a commercial phase noise analyser (PNA, Rohde & Schwarz FSWP50). The PSD of the frequency noise $S_{\nu}(f)$ can be directly derived form $S_{\Delta\phi}(f)$ by[7]

$$S_{\nu}(f) = \frac{f^2}{4\left[\sin\left(\pi f \tau_{\mathrm{d}}\right)\right]^2} S_{\Delta\phi}(f), \tag{S5}$$

where f is the Fourier frequency offset, ν is the laser frequency. The intrinsic linewidth $\Delta\nu_{\mathrm{intrinsic}}$ of the laser can be obtained from the white frequency noise in the high Fourier frequency offset by $\Delta\nu_{\mathrm{intrinsic}} = 2\pi S_{\nu}^{0}$, where S_{ν}^{0} is the single-sideband PSD of the white frequency noise[7,8].

Allan deviation of the DFB laser frequency is measured by the PNA after beating it with a fiber laser (Koheras

ADJUSTIK E15) which is stable enough compared to the DFB laser in SIL, as shown in Fig. S6b. Also the Allan deviation of the platicon induced microwave is measured by the PNA.

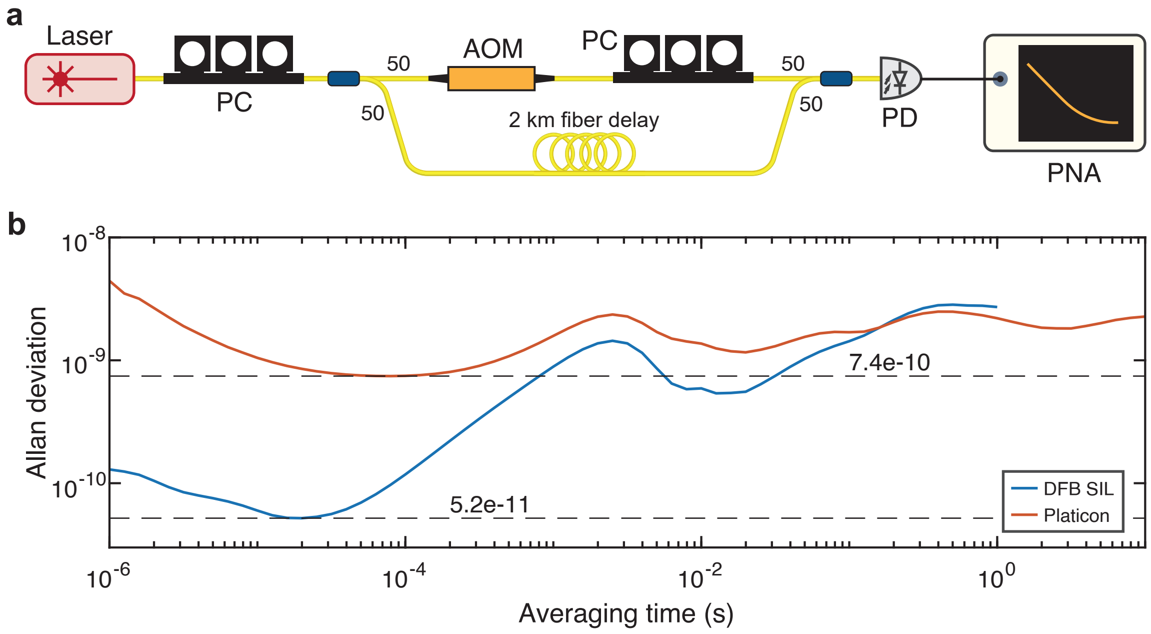

Figure S6: DSHI and Allan deviation. **a**. Experimental setup. PC, polarization controller. AOM, acousto-optic modulator with a center modulation frequency of 80 MHz. PD, photodetecter, Finisar XPDV3120R. PNA, phase noise analyser, R&D FSWP50. **b**. Allan deviation of SIL DFB laser frequency (blue line) and platicon induced microwave frequency (red line).

Supplementary Note 8. Simulation on platicon generation and switching

To simulate platicon generation, we use the dimensionless coupled mode equations

$$\frac{\partial a_\mu}{\partial \tau} = -(1+\mathrm{i}\zeta_\mu)a_\mu + \mathrm{i}\sum_{\mu'\mu''} a_{\mu'}a_{\mu''}a^*_{\mu'+\mu''-\mu} + \delta_{0\mu}f \tag{S6}$$

where μ is the relative mode number of the optical comb, $a_\mu = \sqrt{g\epsilon_0 n_0^2 V_{\mathrm{eff}}/(\kappa\hbar\omega_0)}E_\mu$ is the dimensionless amplitude of μth mode, $g = \hbar\omega_0^2 c n_2/(n_0^2 V_{\mathrm{eff}})$ is the Kerr coefficient, V_{eff} is the effective mode volume, n_0 is the refractive index of the waveguide, ω_0 is the central mode frequency, $\tau = \kappa t/2$ is the dimensionless time, $f = \sqrt{8g\kappa_{\mathrm{ex}}P_{\mathrm{in}}/\kappa^3}$ is the dimensionless pump amplitude, P_{in} is the pump power, $\zeta_\mu = -\xi + d_2\mu^2/2 - \Delta_\mu$, $\xi = 2(\omega_{\mathrm{p}}-\omega_0)/\kappa$ is the dimensionless detuning, $d_2 = 2D_2/\kappa$ is the dimensionless second order dispersion coefficient, Δ_μ is the dimensionless eigenfrequency shift of μth mode which is caused by self-injection locking[9]. In our simulation, we set $d_2 = -0.0096$ ($D_2/2\pi = -87.27$ kHz and $\kappa = 2\pi \times 17.6$ MHz), $f = 3.243$ ($P_{\mathrm{in}} = 98$ mW), $V_{\mathrm{eff}} = 4.58 \times 10^{-14}$ m^3, $\Delta_0 = 1.107$, $\Delta_{\pm 2} = \mp 0.0615$, $\Delta_{\pm 3} = \pm 0.0123$, $\Delta_{\pm 6} = \mp 0.123$. The intra-cavity power evolution is shown in Fig. S7a. By tuning the laser frequency from blue detuning to red detuning, stable platicon state is excited in the cavity and the optical power suddenly drops down to a relatively low value. Then tuning the laser frequency back, discrete platicon power steps can be observed. The platicon waveforms at different descrete states are shown in Fig. S7b.

In order to calculate the change of the repetition rate of platicon, we use $a(\tau,\phi) = \mathcal{F}[a_\mu](\tau,\phi) = \sum_\mu a_\mu(\tau)\mathrm{e}^{\mathrm{i}\mu\phi}$ to transform the coupled mode equations to a modified LLE

$$\frac{\partial a}{\partial \tau} = -a - \mathrm{i}\mathcal{F}[\zeta_\mu a_\mu](\tau,\phi) + \mathrm{i}|a|^2 a + f \tag{S7}$$

where ϕ is the azimuthal angle of the rotating coordinate system. Then we assume that the deviation of the platicon repetition rate from the FSR is V[10], meaning that it runs at a speed of V in the rotating coordinate system. So we can transform the present coordinates τ, ϕ to new coordinates τ', ϕ' with $\phi' = \phi - V\tau$, $\tau' = \tau$. Clearly, in this new coordinate system the platicon waveform along the azimuthal angle is stationary, and the modified LLE becomes

$$\frac{\partial a}{\partial \tau'} = -a + V\frac{\partial a}{\partial \phi'} - \mathrm{i}\mathcal{F}[\zeta_\mu a_\mu](\tau',\phi'+V\tau') + \mathrm{i}|a|^2 a + f \tag{S8}$$

Because the platicon in this coordinate system is stationary, so the left side of above equation equals zero, and then we can use the right side of the equation to get the repetition rate deviation V from the FSR. The repetition rates at different conditions of modes shifting are shown in Fig. S7c.

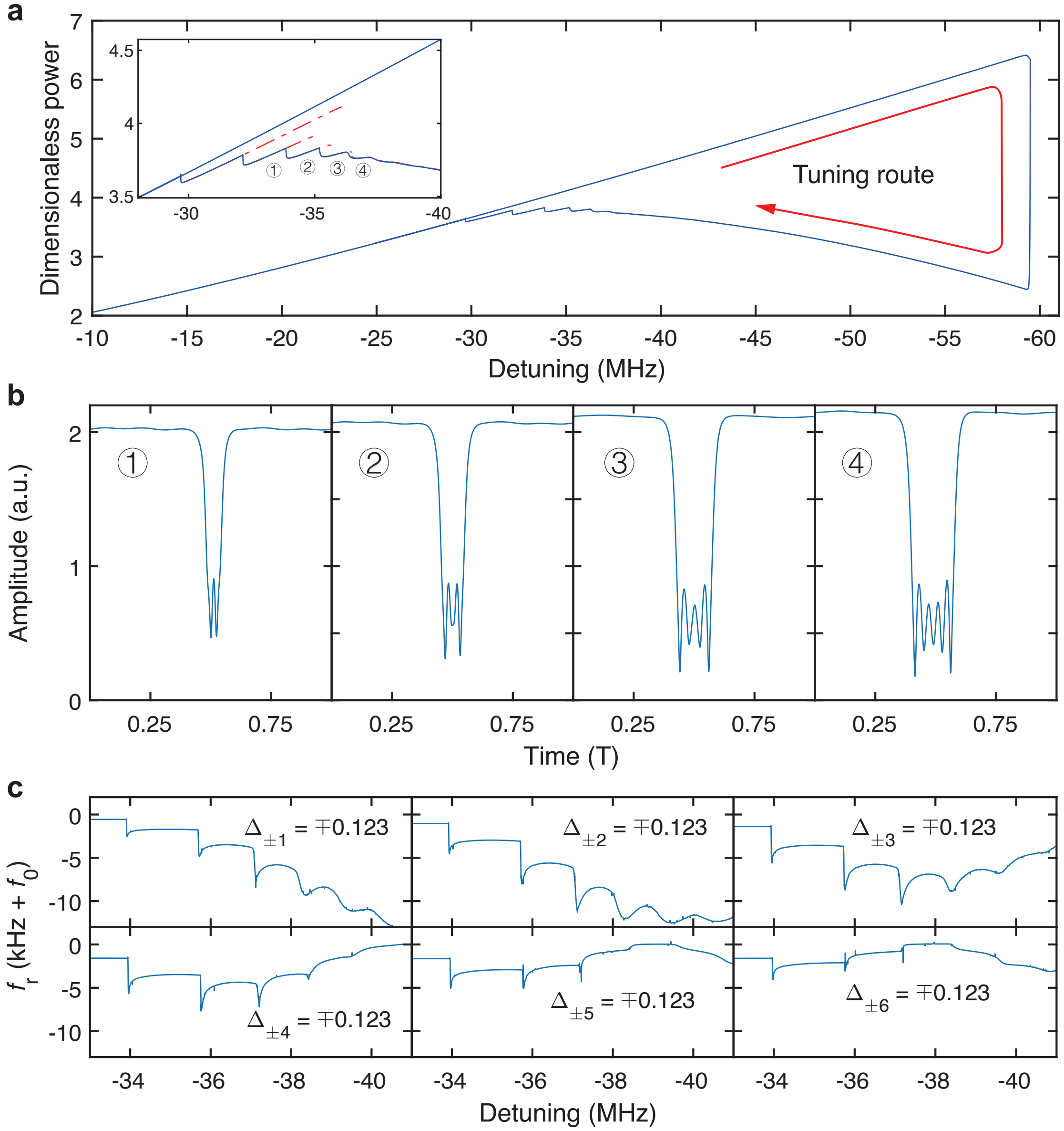

Figure S7: Simulation on platicon generation. **a**. The blue line represents the intra-cavity optical power. The arrowed red line represents the laser tuning route. The inset is zoom-in view on the optical power where the dot-dashed lines represent the complete stable-state power distribution. **b**. Waveform in time domain. T represents the rountrip time. The numbers represent the different platicon states as the same as that in the main text. **c**. Repetition rate under different combinations of modes shiftings. $f_0 = 10.685550$ GHz is a frequency offset equal to the FSR or $D_1/2\pi$ of the microresonator.

Supplementary Note 9. Comparison with commercial microwave oscillators

Here we compare the phase noise and size of our microcomb-based photonic microwave oscillator with those of commercial microwave oscillators, as shown in Fig. S8. The phase noise data correspond to microwave carrier frequency scaled to 10 GHz in the X band. Commercial oscillators operating in the microwave X band are commonly based on dielectric resonator oscillators (DROs) or optoelectronic oscillators (OEOs). Typically, the phase noise is higher for higher microwave carrier frequency. As evidenced in Fig. S8, our hybrid photonic microwave chip has small size and competitive phase noise performance.

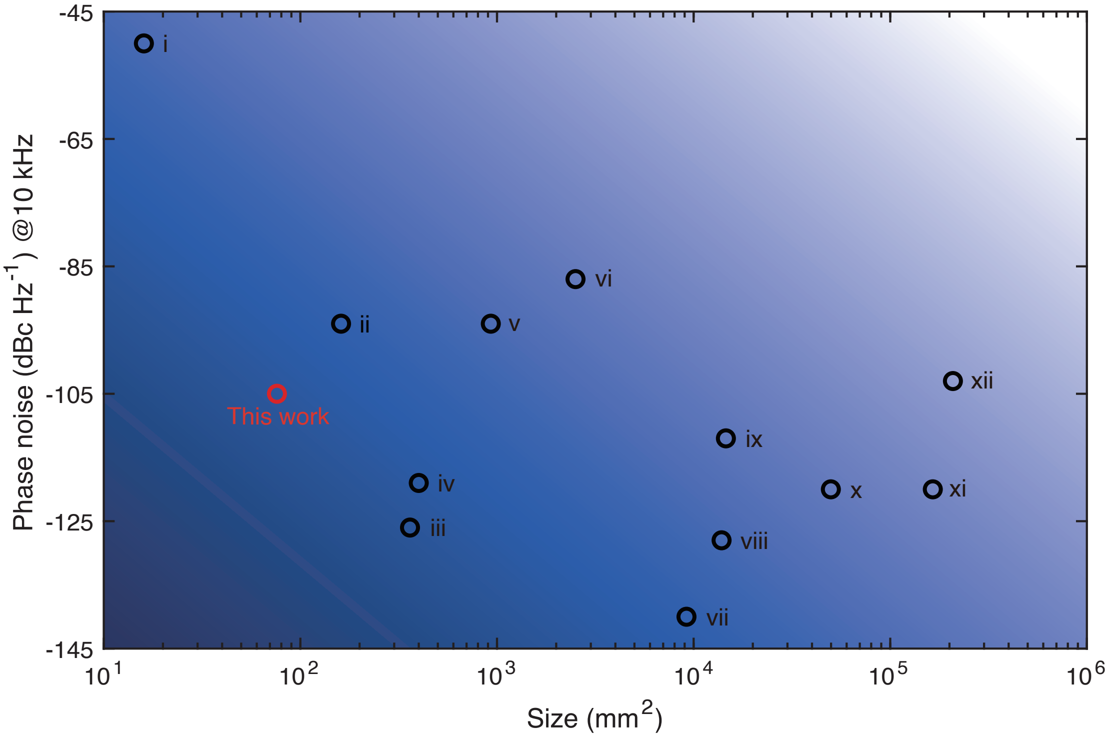

Figure S8: Comparison on the phase noise and size of different microwave oscillators. The vertical axis is the phase noise at 10 kHz Fourier frequency offset. The horizontal axis is the device size. The color gradient indicates the combined performance of phase noise and size, where the dark zone marks low phase noise and small size. The red circle marks the performance of our comb-based photonic microwave oscillator. The black circles mark the performance of: i. Analog Devices ADF5709, ii. Mini-Circuits ROS-6840C-199+, iii. Synergy GSDRO1000-8XT, iv. OEwaves HI-Q Nano OEO, v. Quantic MWD DRO-1500, vi. Narda-MITEQ DRO-G-10000, vii. Rakon LNO10000B3, viii. Quantic Wenzel MXO-PLM, ix.Holzworth HSM12001B, x. AnaPico APULN, xi. Holzworth HSY9000, xii. Keysight N5173B. Note that all the phase noise data are compared with microwave carrier frequency scaled to 10 GHz.

Supplementary References

[*] These authors contributed equally to this work.
[†] chenbl@shanghaitech.edu.cn
[‡] liujq@iqasz.cn

[1] Ye, Z. *et al.* Foundry manufacturing of tight-confinement, dispersion-engineered, ultralow-loss silicon nitride photonic integrated circuits. *Photon. Res.* **11**, 558–568 (2023). URL https://opg.optica.org/prj/abstract.cfm?URI=prj-11-4-558.

[2] Luo, Y.-H. *et al.* A wideband, high-resolution vector spectrum analyzer for integrated photonics. *Light: Science & Applications* **13**, 83 (2024). URL https://doi.org/10.1038/s41377-024-01435-z.

[3] Kondratiev, N. M. *et al.* Self-injection locking of a laser diode to a high-Q WGM microresonator. *Opt. Express* **25**, 28167–28178 (2017). URL https://opg.optica.org/oe/abstract.cfm?URI=oe-25-23-28167.

[4] Kondratiev, N. M. *et al.* Recent advances in laser self-injection locking to high-Q microresonators. *Frontiers of Physics* **18**, 21305 (2023). URL https://doi.org/10.1007/s11467-022-1245-3.

[5] Coldren, L., Corzine, S. & Mashanovitch, M. *Diode Lasers and Photonic Integrated Circuits*. Wiley Series in Microwave and Optical Engineering (Wiley, 2012). URL https://books.google.ch/books?id=GBB1k0YONT4C.

[6] Okoshi, T. & Kikuchi, K. *Coherent optical fiber communications*, vol. 4 (Springer Science & Business Media, 1988).

[7] Camatel, S. & Ferrero, V. Narrow linewidth CW laser phase noise characterization methods for coherent transmission system applications. *Journal of Lightwave Technology* **26**, 3048–3055 (2008). URL https://ieeexplore.ieee.org/document/4738475.

[8] Jin, W. *et al.* Hertz-linewidth semiconductor lasers using CMOS-ready ultra-high-Q microresonators. *Nature Photonics* **15**, 346–353 (2021). URL https://doi.org/10.1038/s41566-021-00761-7.

[9] Lobanov, V., Lihachev, G., Kippenberg, T. J. & Gorodetsky, M. Frequency combs and platicons in optical microresonators with normal GVD. *Opt. Express* **23**, 7713–7721 (2015). URL http://www.opticsexpress.org/abstract.cfm?URI=

oe-23-6-7713.

[10] Lobanov, V. E. *et al.* Fragmentation of stability domains of dark solitons, dark breathers, and drifting solitons at high pump intensities in normal-dispersion Kerr microresonators. *Phys. Rev. A* **109**, 033524 (2024). URL https://link.aps.org/doi/10.1103/PhysRevA.109.033524.
